# TH1 cytokine response to HCV peptides in Egyptian health care workers: a pilot study

**DOI:** 10.1186/1743-422X-10-144

**Published:** 2013-05-11

**Authors:** Mona M Rafik, Alaa El-Dien MS Hosny, Khaled O Abdallah, Amal A Abbas, Rania A Abo Shady, Dina A Soliman, Khaled M Nasr El-Din Rakha, Shahira F Alfedawy

**Affiliations:** 1 Ain Shams Faculty of Medicine Clinical Pathology Department, Abbassia square, Cairo, Egypt; 2 Cairo Faculty of Medicine Department of Microbiology and Immunology, Kasr El Ainy St, Cairo, Egypt

**Keywords:** CFSE, Proliferation assay

## Abstract

Our objective was to elucidate the effects of different HCV peptides on TH1 cytokine synthesis (interleukin 2(IL2), gamma interferon (INFγ) and tumor necrosis factor α (TNF α)), in a proliferative response in a high risk population of HCV seronegative aviremic Egyptian healthcare workers (HCW). We studied the TH1 cytokine response to different HCV peptides among 47 HCW with and without evidence of HCV infection. Participants were classified according to the proliferation index (PI) in a CFSE proliferation assay as an indicator of previous exposure to HCV. Cytokines were analyzed using Luminex xMAP technology. Results showed that positive PI HCW produced a higher IL2 in response to all HCV peptides except NS4, a higher IFNγ response to NS3 and NS4 and no difference in TNFα response when compared to the negative PI HCWs. When compared to chronic HCV HCW, positive PI HCW showed no difference in the IL2 response, a higher IFNγ response to NS4 and NS5 HCV peptides and a higher TNFα response to all peptides. In conclusion the magnitude and type of cytokines produced in HCV infection is critical in determining the outcome of infection. NS4 & NS5 HCV peptides induce a protective TH1 response in positive PI HCW

## Background

Prospective studies of healthcare workers (HCW) after occupational exposure have identified the average hepatitis C virus (HCV) transmission rate associated with contaminated needle sticks to be 1.8% [1]. Kubitschke et al. 2007 carried out a systematic review of 22 studies reporting a total of 6956 injuries involving HCV-contaminated needles and demonstrated seroconversion rates ranging between 0% and 10.3%, with a mean of 0.75% with worldwide differences in HCV seroconversion rates. This suggests that genetic factors might provide some level of natural resistance against HCV [2]. Little is known about the prevalence of HCV among HCW in Egypt, where the highest worldwide prevalence of HCV exists. In recent studies the prevalence of HCV antibody in Egyptian HCW workers was found to range from 11.2% to 16.6% [3,4] and HCV viraemia was found to be 8%, but the prevalence was not significantly different than that found among non-medical personnel. Previous studies in Egypt concluded that the infections were more likely to be community-acquired and not occupationally related but Rafik et. al [3] added that workers in the dialysis unit and laboratory technicians showed significantly higher prevalence than other job groups in the same departments.

HCV-specific T-cell responses have been described in studies of exposed HCV-seronegative individuals [2-5] where these responses were found to be broad and associated with risk factors for HCV exposure, suggesting that they reflected true exposure to HCV in seronegative persons. Evidence for the presence of a cellular immune response was detected by a CFSE assay where a positive proliferative response to different HCV peptides was detected in 27/60 aviraemic seronegative Egyptian laboratory and blood bank HCW [6].

Conflicting results have been reported about the magnitude, breadth, quality and duration of HCV specific immune responses in different clinical settings of HCV infection. Due to the difficulty in identifying persons who successfully clear acute HCV; examination of immune responses in large numbers of exposed but uninfected persons provides an alternate strategy for identification of protective immune responses in HCV. Our aim was thus to research one aspect of the protective HCV immune response in HCW, the TH1cytokine response to different HCV peptides in a high risk population of Egyptian HCW, to elucidate the effects of different HCV peptides on cytokine synthesis in a proliferative response. The hypothesis was that the type and level of TH1 cytokines produced during their HCV exposure may be a critical determinant of viral clearance

## Material and methods

The study began with screening 240 of the staff working in Ain shams University Hospitals (Internal Medicine Hospital). The study sample was composed of different categories of professionals. One hundred eighty seven health care workers (personnel who either had direct contact with patients or with potentially infectious clinical specimens as blood and body fluids.) were enrolled in the study and included 48 resident doctors, 77 nurses, 17 technicians and 45 workers. A control group was included; consisting of 53 of the administrative employees (secretaries, personnel management employees, accounting employees, etc.…) in the same hospital who had no contact with patients, or with material used by them.

After written consent a detailed questionnaire was obtained from each participant with special emphasis on occupational risk factors for HCV which included duration of employment, exposure to needle stick injuries, accidental splashes of blood or body fluids onto mucous membranes and failure to adhere to essential infection control procedures (gloves). All participants were initially tested for ALT level, and HCV-antibody carried out by third-generation enzyme-linked immunosorbent assay (ELISA) for serum anti-HCV antibodies. HCV RNA detection was carried out by Real-time RT-PCR for all participants:

The study showed that according to the occupation laboratory technicians had a greater prevalence of infection than other occupations (p < 0.01) in different departments of the hospital.

Among the 187 HCWs included in the study, 67.9% reported exposure to needle stick injuries, 56.7% reported splashes of blood or body fluids to the eye or mucous membranes, 82.4% handled body fluids as part of their job description, 17.6% did not use gloves, 53.5% used gloves and 28.9% used gloves intermittently. HCV infection was statistically significantly associated with failure to use gloves (p < 0.05).

After determining the HCV status of the previous subjects, 60 were selected from the laboratory and blood bank personnel since they had the greater prevalence of infection than all other HCW and the highest exposure to blood (Table 1). They included 60 HCWs (38 males and 22 females) with a mean age of 36 ± 10 years who worked at Ain Shams University laboratory and blood bank. Only 10 of them were found to be viraemic (HCV PCR + ve) and were HCV antibody + ve while all the other were seronegative for HCV antibody and aviraemic for HCV.

**Table 1:** Demographic characteristics of HCWs

**Characteristic**	**Number (Total = 60)**	**Percentage**
***Gender***		
Female	22	36.7%
Male	38	63.3%
***Age (years)***		
< 25	5	8.4%
25–40	43	71.7%
> 40	12	19.9%
***HCV RT-PCR***		
Positive	10	16.7%
Negative	50	83.3%
***Work place***		
Laboratory	45	75%
Blood Bank	15	25%
***Job category***		
Worker	15	25%
Resident physician	13	21.7%
Nurse	7	11.7%
Technician	25	41.7%
***Prick injury***		
Positive history	31	51.7%
Negative history	29	48.3%
***Family history***		
Positive history	14	23.3%
Negative history	46	76.7%

PBMCs from study subjects were isolated immediately from fresh heparinized blood by Ficoll-Hypaque density gradient centrifugation using standard techniques and immediately tested for antigen-specific cell proliferation and CFSE assays [7]. Proliferation assays were performed without or after 5, 6-carboxyfluorescein diacetate succinimidyl ester.

Freshly isolated cells were resuspended in PBS that had been supplemented with 5% FCS (Eurobio), to a final cell concentration of 1 million / ml. CFSE was added at a concentration of 5 μM/μL (Molecular probes, Invitrogen). After 10 min incubation, at 37°C, in the dark cells were pelleted, washed extensively, and resuspended in 1 ml of ice cold RPMI-1640 that had been supplemented with 10% FCS, 1% HEPES, 0.2% Gentamicin. CFSE labeled cells were then stimulated in a 7-day culture with (PHA) (Sigma Aldrich) (10 μg /ml) as a positive control and HCV peptides (core (aa 2–120), nonstructural protein 3 (NS3) (1192–1457), nonstructural protein 4 (NS4) (aa 1569–1931), and nonstructural protein 5 (NS5) (aa 2054–2995core,) (Chiron) (4 μg /well) for 7 days in 10% CO2 at 37°C. Unstimulated cells with medium only were used as negative controls.

On the seventh day GUAVA EASY cyte 4 color flow cytometry was used to determine the percent of the parent cells and the mean fluorescence intensity (MFI) of the CFSE stained cells. The Proliferation index (P.I) was calculated according to Lyons and Parish, 1994 [7]. P.I was considered + ve if it was higher than the cut off values calculated (= Mean P.I of positive Control group x1 .68 standard deviation (SD) for each peptides used).

Cell culture supernatants from the peripheral blood mononuclear cells (PBMCs) stimulated by HCV peptides in the CFSE proliferation assay for 47 HCW were collected and stored at −80°C until assayed. The personnel included in this study were divided into two groups:

1) **HCV double negative (HCWs)**

(negative PCR & antibody) (Group I): This group included 37 HCWs who had normal serum alanine aminotransferase, serum aspartate aminotransferase, and negative HCV antibody & were negative for HCV RNA. According to the proliferation index (PI) in the CFSE proliferation assay for the HCV peptides, group 1 HCW was further classified into 2 subgroups.

Group1a) **Positive proliferation index (+ve P.I) HCWs:** Subjects whose cells showed a P.I more than the cut off values of the PI for the HCV specific peptides of the chronic group in the CFSE assay (group 2) (27 samples were available).

Group1a) **Negative proliferation index (−ve P.I) HCWs:** Subjects in group I whose cells showed a P.I less than the cut off values of the PI of HCVspecific peptides of the chronic group in the CFSE assay (10 samples).

2) **Chronic HCV HCWs (positive Control group): (Double Positive: PCR & antibody for HCV)(Group II)) which consisted of 10 HCW**

HCV proteins used were derived from the HCV-genotype 1 sequence and encoded core (aa 2–120), nonstructural protein 3 (NS3) (1192–1457), nonstructural protein 4 (NS4) (aa 1569–1931), and nonstructural protein 5 (NS5) (aa 2054–2995).

### TH1 Cytokine analysis

All cell culture supernatants were analyzed using Luminex xMAP technology for IL2, IFNγ and TNFα. Reagents were supplied by ***R&D diagnostics*** designed for use with the ***Luminex100™****,* Analyzer manufactured by Luminex Corporation. (R&D systems, Minneapolis, United States of America).

All protocols for this study were reviewed and approved by the Ain Shams Faculty of Medicine ethics committee and all participants provided written informed consent.

### Statistical analysis

IBM statistical package for social sciences (SPSS statistics) (V. 19.0, IBM Corp., USA, 2010) was used for data analysis. Data was expressed as median and percentiles for quantitative non-parametric measures utilizing Wilcoxon Rank Sum test, Ranked Spearman correlation test and Kruskall Wallis test

## Results

### Cytokine responses to HCV peptides produce a similar pattern within each individual group

All of the HCV peptides, structural and nonstructural, (Core, NS3, NS4 and NS5) studied produced a similar IL2 and INF γ response with no significant difference between them in each group. However in the positive PI HCW the TNFα response was significantly higher than the other studied groups (Table 2). Correlations between the cytokines studied in response to the different HCV peptides within each group were done and Spearman correlation coefficient (R_s_) is reported (Table 3).

**Table 2:** Cytokine response to different HCV peptides in studied groups (Kruskall Wallis)

**Group**	**Cytokine**	**Median cytokine (pg/ml)**				**p**
		**HCV PEPTIDES**				
		**CORE**	**NS3**	**NS4**	**NS5**	
Positive PI HCW	IL2	16.855	36.18	17.405	20.18	0.209
	IFN-γ	2.16	11.4	3.31	2.0	0.662
	TNFα	60.2	66.4	75.8	117	0.012*
Negative PI HCW	IL2	1.6	6.8	3.7	3.33	0.196
	IFN-γ	.83	1.36	0	0.83	0.588
	TNFα	14.8	157.4	30.4	79.5	0.168
Chronic HCV HCW	IL2	10.9	16.1	8.7	28.33	0.807
	IFN-γ	.065	0.35	0.28	0.175	0.583
	TNFα	16.2	18.0	25. 8	20.3	0.989

p * ≤ 0.05 significant.

**Table 3:** Correlation between cytokine responses to HCV Peptides within individual groups

**Group**	**INFγ & IL2**			**IL2 & TNFα**			**TNFα & INFγ**		
	**HCV Peptide**	**Rs**	**p**	**HCV Peptide**	**Rs**	**p**	**HCV Peptide**	**Rs**	**p**
**1-a:Positive P.I HCWs:**	Core	**0.514**	**0.014**	Core	0.279	0.21	Core	**0.447**	**0.028**
	NS3	**0.51**	**0.024**	NS3	−0.1	0.6	NS3	0.2	0.3
	NS4	0.226	0.311	NS4	0.797	0.44	NS4	**0.436**	**0.026**
	NS5	**0.512**	**0.015**	NS5	0.127	0.60	NS5	**0.445**	**0.03**
**1-b: Negative P.I HCWs:**	Core	0.403	0.282	Core	−0.53	0.14	Core	−0.153	0.694
	NS3	0.451	0.223	NS3	−0.6	0.1	NS3	−0.5	0.1
	NS4	**0.893**	**0.003**	NS4	0.47	0.24	NS4	−0.035	0.924
	NS5	**0.72**	**0.029**	NS5	0.644	0.06	NS5	0.338	0.339
**2-Chronic HCV HCW**	Core	−0.395	0.439	Core	−0.6	0.28	Core	−0.112	0.858
	NS3	**−0.894**	***0.041***	NS3	−0.1	0.8	NS3	**0.8**	**0.04**
	NS4	0.051	0.935	NS4	0.60	0.285	NS4	0.698	0.123
	NS5	−0.213	0.686	NS5	−0.5	0.391	NS5	0.759	0.08

Spearman correlation coefficient (R_s_) is reported.

Strong correlations (|R_s_| ≥ 0.5) are indicated in bolded print; **black** indicates positive correlations; **italics** indicates negative correlations.

In positive PI HCW both structural (core) and nonstructural (NS3 and NS5) HCV peptides produced a positive correlation between IL2 and INFγ while in the negative HCW only nonstructural peptides (NS4 & NS5) did. There was no correlation between IL2 and TNF produced in response to HCV peptides in all studied groups. There was a positive correlation between INFγ and TNFα produced in response to core NS4 and NS5 in positive PI HCW and to NS3 in chronic HCV HCW

### Differentiating characteristics of the cytokine response between groups

There was no difference in IL2 response to all the HCV peptides between positive HCW and the chronic HCV HCWs group (Table 4) but there was a higher IL2 response to all the HCV peptides except NS4 in comparison to the negative HCW Only the core peptide resulted in a higher IL2 response in the chronic HCV HCW compared to the negative PI HCW.

**Table 4:** Comparison between the IL2 responses to different HCV peptides within the studied groups

**HCV peptide**	**Groups tested**								
	**Positive P.I HCWs**	**Chronic HCV HCW**	**p**	**Positive P.I HCWs**	**Negative P.I HCWs**	**p**	**Chronic HCV HCW**	**Negative P.I HCWs**	**p**
	**Median IL2 (pg/ml)**			**Median IL2 (pg/ml)**			**Median IL2 (pg/ml)**		
CORE	16.85	10.995	0.575	16.85	1.6	**0.006**	10.995	1.6	**0.009**
NS3	36.18	16.1	0.165	36.18	6.82	**0.013**	16.11	6.82	0.461
NS4	17.40	8.7	0.574	17.40	3.69	0.071	8.7	3.69	0.305
NS5	20.18	28..33	0.674	20.18	3.33	**0.046**	28.33	3.33	0.093

Significant difference (Wilson Rank Sum) (p ≤ 0.5) is indicated in bolded **black** print.

Regarding the IFNγ response in positive HCW only NS4 and NS5 peptides resulted in a significantly stronger IFNγ response compared to chronic HCV HCWs and a higher response to NS3and NS4 peptides compared to the negative HCW group (Table 5).

**Table 5:** Comparison between INFγ responses to different HCV peptides within the studied groups

**HCV peptide**	**Group tested**								
	**Positive P.I HCWs**	**Chronic HCV HCW**	**p**	**Positive P.I HCWs**	**Negative P.I HCWs**	**p**	**Chronic HCV HCW**	**Negative P.I HCWs**	**p**
	**Median INFγ (pg/ml)**			**Median INFγ (pg/ml)**			**Median INFγ (pg/ml)**		
CORE	2.16	0.065	0.103	2.16	0.83	0.119	0.065	0.83	0.376
NS3	11.4	0.35	0.055	11.4	1.36	**0.031**	0.35	1.36	1
NS4	3.31	0.28	**0.026**	3.31	0	**0.004**	0.28	**0**	0.811
NS5	2	0.2	**0.041**	2	1	0.135	0.2	1	0.295

Significant difference (Wilson Rank Sum) (p ≤ 0.5) is indicated in bolded **black** print.

The IFN gamma response to NS4 in positive HCW is significantly higher than in negative HCW whereas there was no significant difference in the IL2 response to this peptide. There was no correlation between both cytokines in response to NS4 in positive HCW and there was such a correlation in the negative HCW.

In this study NS3 peptide evoked the strongest INFγ response in all groups but this was not significantly different except in the positive HCW who were higher than the negative HCW. The IL2 response to the NS3 peptide in the positive HCW was higher than that in the negative HCW with the presence of a positive correlation with IFN γ which could not be detected in the negative HCW. The HCV NS3 peptide seems to stimulate a predominantly IL2 dependent INFγ production in positive PI HCW.

The TNFα response to the different HCV peptides showed no difference between positive and negative PI HCW with a significantly higher response to all peptides between positive PI HCW and chronic HCV HCW. NS3 and NS5 peptides produced a lower TNFα in chronic HCV HCW than even the negative PI HCW (Table 6).

**Table 6:** Comparison between the TNFα response to different HCV peptides within the studied groups

**HCV peptide**	**Groups tested**								
	**Positive P.I HCWs**	**Chronic HCV HCW**	**p**	**Positive P.I HCWs**	**Negative P.I HCWs**	**p**	**Chronic HCV HCWs**	**Negative P.I HCWs**	**p**
	**Median TNFα (pg/ml)**			**Median TNFα (pg/ml)**			**Median TNFα (pg/ml)**		
**CORE**	60.18	16.17	**0.026**	60.18	14.81	0.063	16.17	14.81	0.841
**NS3**	66.4	18	**0.002**	66.4	157.4	0.678	18	157.4	**0.007**
**NS4**	75.8	25.8	**0.002**	75.8	30.4	0.374	25.8	30.4	0.277
**NS5**	117	20.3	**0.000**	117	79.5	0.112	20.3	79.5	**0.017**

Significant difference (Wilson Rank Sum) (p ≤ 0.5) is indicated in bolded **black** print.

The NS4 and NS5 peptide results typically display a type 1 TH cytokine profile in positive PI HCW which is not true in chronic HCV HCW where they induce a lower INFγ and TNFα response.

## Discussion

Clearance of HCV-RNA may not necessarily correlate with the appearance of acquired immunity and may be an explanation for the rather low seroconversion rate after occupational exposure to HCV [8]_._ Studies indicate that selected high-risk subjects can mount an effective immune response against HCV infection in the absence of antibody production and that they may have a higher likelihood of viral clearance in that setting [9-14].

The immunodominance hierarchies within HLA-A2-restricted HCV-specific CD8+ T cell responses were recently studied to determine if they are linked to naive-precursor frequency [15]. Precursor frequencies to Core132, NS3 1406 and NS5B 2594 specific epitopes were conserved across the healthy donors although they varied significantly between epitopes suggesting that a clear hierarchy of naive HCV-specific CD8 + T cell precursor frequencies exists in healthy donors [15]. The NS3 1406 epitope had the highest naive-precursor frequency in healthy donors and the most frequently targeted epitope in chronic HCV-infected patients, both *ex vivo* and after *in vitro* stimulation, whereas the core 132 epitope was rarely targeted in most studies [16-19]. For assessing human antiviral T cell responses the functions quantified simultaneously include up regulation of IFN γ and IL2 [20-22]. In this study of all the HCV peptides studied NS3 peptide (1192 to 1457) evoked the highest IL2 response in both positive and negative HCW whereas the core peptide produced the lowest IL2 response. The difference in the IL2 induced was significantly higher in positive HCW than the negative HCW for all peptides except NS4. The most predominant IL2 response in chronic HCV HCW was obtained by NS5 and the lowest was by the NS4 peptide but the only significantly higher IL2 response compared to the negative HCW was that obtained by the core peptides. When compared to the positive HCW, all the HCV peptides in chronic HCV HCW produced lower levels of IL2 but with no significant difference.

IL2 plays a critical role in the regulation of Th1 and Th2 responses and impacts INFγ production [23]. INFγ production in chronic HCV HCW was not significantly different from the negative PI HCW for all peptides tested. At the same time positive PI HCW showed a significantly higher INFγ in response to NS4 and NS5 than the chronic HCV HCW and a significantly higher INFγ in response to NS3 and NS4 than the negative HCW. The failure to produce INFγ, in response to HCV NS4 peptide in chronically HCV-infected patients has been previously documented [24].

IL2 cytokine secretion in response to a panel of recombinant HCV antigens in patients with acute HCV has been studied previously and showed that patients with self-limited disease had a significant secretion of IL2 [25] and those who developed chronicity had significantly lower IL-2 [26] and INFγ production than those cases who recovered [27,28]. The previously described ’stunned’ T cell phenotype in chronic HCV infection [20,29] was seen in this study for almost all of the nonstructural peptides (NS4 and NS5) with near significance for NS3 (p = 0.055). Resolved HCV infection is associated with T-cells responses with higher IFNγ predominantly targeting nonstructural proteins [30-34]. The lowest magnitude of responses in persons with resolved infection was to the relatively small core protein, and these responses were not significantly different from persons with chronic infection [35]. Perrella et al. (2009) [36] searched for evidence of hepatitis C virus-specific interferon gamma-positive T cells in health care workers in an infectious disease department. They found that levels of HCV-specific IFN-gamma-positive cells were higher in the HCWs compared with the infected patients and healthy blood donors. The investigators concluded that a clinically silent persistent exposure to HCV, through some as-yet undetermined mechanism, may induce a virus-specific IFN-gamma-producing CD8 (+) T-cell response in healthy aviremic HCWs. This finding suggests that possible unapparent parenteral routes may stimulate host defenses with no evidence of hepatitis.

The observed pattern of a vigorous CTL response and relatively weaker humoral response predicting disease resolution suggests that HCV persistence also relates to the pattern of cytokine release [14]. Persistence may relate to a relative insensitivity of HCV to antiviral cytokines such as TNFα and IFNγ [10,18],since viral clearance was associated with a greater magnitude and broader specificity of IFNγ and IL2 responses [37]. Finally the frequent and low levels of HCV viremia may have been controlled by innate immune responses without a strong adaptive immunity [8].

Cross-reactivity to other commonly encountered antigens is an alternative explanation for the T cell responses found However, a range of viral proteins elicited responses, including the structural proteins core as well as the nonstructural proteins, with no single antigen standing out as immunodominant. These T cell responses suggest that our subjects were indeed exposed to HCV.

Cell-mediated immune (CMI) responses to hepatitis C virus (HCV) antigens in adults without seroconversion or viraemia are biomarkers for prior transient infection [14]. It may be stipulated that these CMI-positive HCV seronegative subjects may have had a transient very mild infection, possibly associated with low-dose exposure to the virus, which was cleared. Conventional mechanisms for sustaining T cell memory are likely to be involved, including cytokine-driven homeostatic proliferation [38]*.*

NS4 and NS5 protein antigens represent immunogenic, possibly protective, regions of the HCV genome in this group of positive PI HCW. The protective nature of NS5 peptide has been previously reported in uninfected siblings of HCV chronically infected children [14].

The HCV-specific cytokine responses observed in aviremic, seronegative HCW were qualitatively and quantitatively different from those observed in chronic HCV HCW. Our results show that T-helper 1 type cytokines are produced following stimulation with nonstructural antigens (NS4, and NS5) by positive PI HCW (who had resolved HCV infection) similar to the results demonstrated by others [39,40].

Abdulwahab et al. (2012) demonstrated a strong and broad HCV-specific IFN-γ response without detectable viremia or seroconversion in more than half (55.8%) of Egyptian HCW, with both CD4+ and CD4− T cells producing IFN-γ (the highest responses were to NS3 & NS5a). They did not find a significant correlation between the strength and breadth of the HCV-specific CMI in these seronegative aviremic subjects and their demographic, clinical, or laboratory data [41]. The latest research on Egyptian HCW conducted at Ain Shams University Hospital, Cairo included HCWs reporting occupational blood exposure who had neither anti-HCV antibodies (anti-HCV) nor HCV RNA, and were exposed to a HCV RNA positive patient. They were enrolled in a 6-month prospective cohort with follow-up visits at weeks 2, 4, 8, 12 and 24. During follow-up, anti-HCV, HCV RNA and ALT were tested. Of 73 HCWs exposed to HCV RNA from index patients, nine presented transient viremia, the majority of which occurred within the first two weeks after exposure. None of the workers presented seroconversion or elevation of ALT. The authors concluded that HCWs of a general University hospital in Cairo were exposed to a highly viremic patient population and they experienced frequent occupational blood exposures, particularly in early stages of training. These exposures resulted in transient viremic episodes without established infection [42].

The cytokine response against viral protein antigens shown in our study was elicited when PBMCs were cocultured with recombinant HCV antigens without the addition of any particular cytokines in vitro for differentiation into effector cells. Immunological memory in these HCW may have been established upon a prior exposure to the virus and a subsequent exposure to the antigen was capable of inducing a prompt HCV-specific effector function ex-vivo, possibly providing antiviral protection. The maintenance of these effector T cell responses may be sustained by repeated low-level exposure to HCV and stimulation of memory cells.

## Conclusion

In conclusion, we present our observation that a substantial percentage of HCV-seronegative aviremic HCW who are potentially exposed to the virus by virtue of their occupation develop a protective TH1 cytokine profile which may have protected them from persistent infection and probably protects against subsequent exposure. An increased understanding of the cytokine environment early in HCV infection is important for the development of novel therapeutics to facilitate viral clearance.

## Competing interests

The authors declare that they have no competing interests.

## Authors’ contribution

MR designed data collection tools, monitored data collection for the whole research, interpreted the data and drafted and revised the paper. She is guarantor. AH analyzed the data, and drafted the paper. KA carried out the laboratory analysis and analyzed it. AA supervised the laboratory tests and drafted the article and revised it. RA monitored data collection, and revised the draftpaper. DS monitored data collection and laboratory analysis and revised the draft paper. KR carried out the laboratory analysis. SE revised the paper critically. All authors read and approved the final manuscript.
